# Frequency of Congenital Heart Diseases in Prelingual Sensory-Neural Deaf Children

**Published:** 2016-03

**Authors:** Masoud Motasaddi Zarandy, Mohammad Jafar Mahmoudi, Iran Malekzadeh, Sevil Nasirmohtaram

**Affiliations:** 1 *Otorhinolaryngology Research Center, Tehran University of Medical Science, Tehran ,Iran.*; 2 *Department of Cardiology, Amiralam Hospital, Professor of Tehran University of Medical Sciences, Tehran, Iran.*; 3 *Department of Pediatrics, Children’s Medical Center, Tehran University of Medical Sciences, Tehran, Iran.*

**Keywords:** Congenital sensory-neural hearing loss, Congenital heart disease, Echocardiography, Electrocardiography

## Abstract

**Introduction:**

Hearing impairment is the most frequent sensorial congenital defect in newborns and has increased to 2–4 cases per 1,000 live births. Sensory-neural hearing loss (SNHL) accounts for more than 90% of all hearing loss. This disorder is associated with other congenital disorders such as renal, skeletal, ocular, and cardiac disorders. Given that congenital heart diseases are life-threatening, we decided to study the frequency of congenital heart diseases in children with congenital sensory-neural deafness.

**Materials and Methods:**

All children who had undergone cochlear implantation surgery due to SNHL and who had attended our hospital for speech therapy during 2008–2011 were evaluated by Doppler echocardiography.

**Results:**

Thirty-one children (15 boys and 16 girls) with a mean age of 55.70 months were examined, and underwent electrocardiography (ECG) and echocardiography. None of the children had any signs of heart problems in their medical records. Most of their heart examinations were normal, one patient had expiratory wheeze, four (12%) had mid-systolic click, and four (12%) had an intensified S1 sound. In echocardiography, 15 children (46%) had mitral valve prolapse (MVP) and two (6%) had minimal mitral regurgitation (MR). Mean ejection fraction (EF) was 69% and the mean fractional shortening (FS) was 38%.

**Conclusion:**

This study indicates the need for echocardiography and heart examinations in children with SNHL.

## Introduction

Hearing impairment is the most frequent congenital sensorial defect in newborns (1). In developed countries, 2–4 babies per 1,000 live births are born annually with sensorineural hearing impairment, and this range may extend to six per 1,000 live births in developing countries (2,3). In countries with routine consanguineous marriages, congenital deafness is more common. For example, in Jordan, sensory-neural hearing loss (SNHL) occurs in 9–18 babies per 1,000 live births (4).

Deaf children may have many problems in their social lives, including language learning and communication, and these problems may affect a child’s development and prospects in terms of marriage and employment (5). Therefore, deafness creates a considerable economic burden in society. In an Italian research project in 2007, the mean lifetime cost for a subject affected by profound prelingual deafness was assessed at €737,994.76 for a male and €755,404.02 for a female; 4% of which is spent on the treating procedures while 96% is accounted for by educational classes (6).

In total, 90% of all kinds of hearing loss are sensory-neural (7). Approximately 60% of congenital hearing losses are genetic and about 40% are due to environmental factors. SNHL can appear as an isolated finding or as part of a syndrome. In contrast, 30% of genetic hearing losses are syndromic and about 70% are non-syndromic (8,9).

In addition to SNHL, syndromic cases suffer from skeletal, cardiac, renal, and ocular anomalies that may be life-threatening. According to medical research, morphologic abnormalities of the ear, face, or other organs may indicate a recognizable syndrome, and the number of nonsyndormic hearing impairments converting into syndromic impairments constantly increases (10-12). Caignec et al. studied a family with hearing impairment, congenital heart defects, and a congenital opacity of the periphery of the cornea known as embryotaxon. All participants had congenital heart defects, including tetralogy of Fallot, ventricular septal defect, or isolated peripheral pulmonic stenosis. No individual in this family met the diagnostic criteria for any previously described clinical syndrome. Jagged 1 (JAG1) missense mutation (C234Y) in the first cysteine of the first epidermal-growth-factor–like repeat domain of the protein was noted (12).

One anomaly associated with the greatest morbidity and mortality is congenital heart disease. Jervell and Lange-Nielsen (a syndrome which is a cause of congenital profound hearing loss associated with a prolonged QT interval) (13), Noonan, Beckwith-Wiedemann, Waardenburg, and Townes-Brock are the most common syndromes, including congenital heart disease and ear anomalies and deafness. Children presenting for cochlear implantation with these conditions may be asymptomatic, but are at increased risk of sudden death.

Among environmental exposures, congenital rubella syndrome may be the most important, which, aside from hearing impairment, causes congenital cardiac anomalies, deafness, neural, ocular and other organs defects (14). Solorzona-Santos et al. have reported 42 patients younger than 18 months of age with a positive serologic test for immunoglobulin G and M (IgG and IgM) rubella antibodies.

Major manifestations were ocular (74%), neurologic (66%), congenital heart disease (67%), and hearing loss (19%) (15). From an embryological view, the cardiac loop and otic vesicle are both completed by the end of the fourth week (Day 28) (16). These synchronous events could theoretically be the reason for the association between hearing loss and congenital heart disease.

Congenital heart diseases may range from asymptomatic to morbid. Without treatment, complications such as congestive heart failure, hypoxia, polycythemia, cyanosis, and brain abscess may occur and harm the child sooner or later. Nowadays, due to advanced therapeutic and surgical approaches, early diagnosis and timely treatment can prevent complications and offer a nearly normal life to the child.

Although diagnosing congenital heart diseases is possible with non-invasive and simple methods, the lack of symptoms and oversight by the physician and/or parent may cause these diseases to remain undiagnosed, unless investigation of a congenital heart disease is performed due to the presence of other congenital anomalies. As mentioned previously, one of the most common anomalies is deafness, which can be diagnosed in the first few months or years of a child’s life based on the neonatal hearing screening program, a child’s limited reflexes, or the child’s lack of speech.

Because of the high rate of sensory-neural congenital deafness in Iran, we decided to study the number of heart anomalies in children with sensory-neural deafness. 

## Materials and Methods

The subjects for this study were children with sensory-neural deafness who had undergone cochlear implantation and had been referred to our hospital between the years 2008–2011 for speech therapy. The samples were counted and variables such as age, gender, parents’ relationship, family history of congenital heart diseases or congenital deafness were studied. Requirements for this study were: age 10 years or younger (older children were excluded because some congenital heart disorders recover as the child grows older) and the presence of prelingual sensory-neural deafness. After the child’s medical history was taken, he or she was examined by a cardiologist and underwent a 12-lead electrocardiogram. Next, children were assessed using a Doppler echocardiography device (Fukuda Denshi; CF SONIC_UF_ 7700). Based on the Declaration of Helsinki, all ethical factors were considered. All actions were performed with parental consent and kept confidential. 

## Results

Thirty-one children, 15 of whom were boys and of whom 16 were girls, were examined and underwent ECG and echocardiography. The mean age of participants was 55.70 ± 19 months. The youngest child was 30 months of age and the oldest was exactly 10 years old. The mean weight was 16.31 kg, and there were two sets of twins among the children. 

Fourteen children (45%) were born naturally and 11 children (35%) were born by Cesarean section, the information about the other six children (20%) is not available; three children were born pre-term and one was reported to be born after the due date. The mean weight at the time of birth was 2,975 g; three cases had a low birth weight (<2,500 g) and one had a very low birth weight (<1,500 g). Eighteen patients (58%) had no had concomitant disease or history of hospitalization other than sensory-neural deafness and hospitalization for cochlear implantation. Eight patients had suffered from neonatal icterus, three of whom were hospitalized in the neonatal intensive care unit (NICU) and four had abnormalities such as astigmatism, cleft lip, and developmental disorders. In nine cases (29%), the parents were not related prior to marriage and in 13%, the family relationships prior to marriage between the two parents were very distant. 

**Fig1:**
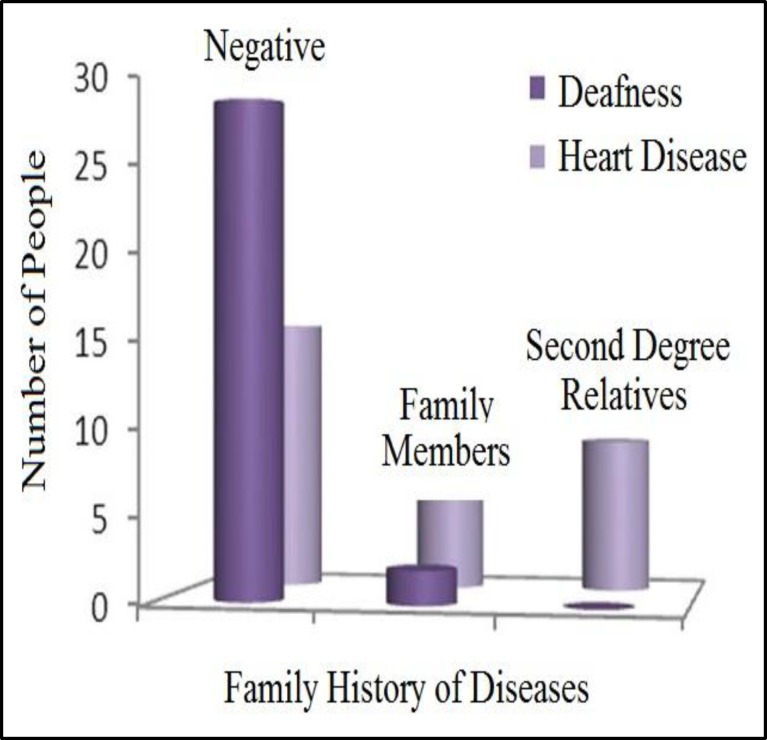
Comparison of the frequency of “Family history of congenital deafness and heart diseases in children with congenital SNHL

Congenital deafness existed in the family history of 13 children (40%). In seven cases (22%), one of the first-degree relatives also had congenital deafness and in six cases, more than one family member had congenital deafness.A family history of congenital heart disease existed in only two cases (6%). In one of these two cases, the mother had mitral valve prolapse (MVP) and in the other, another child in the family had died due to a congenital heart problem. In figure 1, the frequency of family history of congenital deafness and congenital heart disease is presented.

None of the children had any cardiac symptoms in their past medical history. The cardiac examinations of the children were mostly normal. One of the children had expiratory wheezing, four (12%) had mid-systolic click, and four (12%) had intensified S1. In the ECGs of the children, no sign of rhythmic disorder or axis deviation were seen. The mean heart rate of the participants was 123 ± 20 s^−1^ and the mean QTc interval was 418 ± 13 ms. 

In terms of echocardiography, a hyper-dynamic heart was seen in nine children and a reduced myocardial function with normal ventricular diameter (fractional shortening [FS] = 28%, ejection fraction [EF] = 56%) was seen in one child. Fifteen children (46%) had MVP and two (6%) had minimal MR. Mean EF was 68.83% ± 5.8 and mean FS was 37.90% ± 4.4. Table 1 shows the frequency of these data.

**Table 1:** Frequency distribution “Data related to the echocardiography of children with congenital SNHL

**Echocardiography Data**	**Mean**	**Maximum**	**Minimum**	**Standard Deviation**
Rate	123.63	150	83	20.27
QTc (msec)	418.53	474	361	31.08
EF (%)	68.83	82	56	5.89
FS (%)	37.90	48	28	4.44
				

As noted, two sets of twins were present in this study: one set was two homozygote term boys born by Cesarean section (birth weights, 2,500 and 1,800 g, respectively). The second boy suffered from neonatal icterus. The other set was actually triplets, one of whom had died. The two pre-term girls were born by Cesarean section (birth weights, 1,300 and 1,500 g, respectively). Both were admitted to the NICU.

## Discussion

There have been many studies concerning the association of SNHL and congenital heart diseases. For example, Arnold and et al. studied hearing defects in children with congenital heart disease (17), while there are many reports of synchronous anomalies of the ear and heart in an individual or within a family(12,18,19).

In our study, a number of congenital heart diseases were observed in children with sensory-neural deafness. The following points should be noted:

1) Despite the lack of cardiac symptoms, a simple physical exam revealed abnormal cardiac sounds in eight children that required further investigation.

2) According to ECG, the average heart rate of subjects was 123.63 s^−1^ (range, 83–150 s^−1^), which was largely consistent with the values quoted in Nelson’s textbook (120 s^−1^; range, 60–170 s^−1^) (20).

3) The QTc range (corrected QT calculated by $\frac{\mathrm{QT}}{\sqrt[{2}]{\mathrm{RR}}}$) was 361–474 ms. A QTc >450 ms is considered abnormal; in our study five children had a long QT interval. In 2002, a study performed in 52 children with SNHL and 63 children who were healthy was concluded that QTc is prolonged in hearing-impaired children (21).

In a 2003 study by Sopontammarak et al. in 276 children with SNHL, QTs were measured by three pediatric cardiologists and echocardiography was performed in patients with a long QT interval. Two cases of long QT were found and there were no structural abnormalities (22).

The average EF in this study was 69%, with the lowest value of 56% falling within the normal range (55–65%) (23,24). 

The FS is an alternative way of measuring left ventricle performance, and may be a better criterion for studying children’s myocardial functions than EF. FS is calculated according to the formula:

$\frac{\mathrm{LV}\mathrm{end}\mathrm{diastolic}\mathrm{diameter}-\mathrm{LVend}\mathrm{systolic}\mathrm{diameter}}{\mathrm{LV}\mathrm{end}\mathrm{diastolic}\mathrm{diameter}}$

and the normal range is 27–45% (25,26). Only one child had an FS equal to 28%, and the lowest EF (56%) also belonged to this 5-year-old child. However, studies show that EF should be interpreted with caution. 

Fifteen of the children had MVP. The prevalence of MVP is reported as 2.4% in during the Framingham Heart Study (27), but has been quoted to be as low as 1.5% in certain other studies. However, in our studies MVP occurred more frequently. 

MVP is an abnormal movement of one or two mitral leaflets into the left atrium during ventricular systole. Most children with MVP do not need any treatment (28). MVP is generally regarded as a benign condition, but serious complications (including severe mitral insufficiency, cerebral ischemia, infective endocarditis, complex arrhythmias and sudden death) have been described in a minority of patients (29). In our study, three participants suffered from MVP as well as a QT interval longer than 450 ms, and two children had slight mitral regurgitation (MR) and MVP concomitantly. There is a study that shows the prevalence of MR in patients with MVP increased from 29% of the patients to 43% during 4.3 years of follow-up (29).

In addition, in 2009 a group of 10 children, who had been subjected to intense physical training, with silent MVP were examined. A large variety of T-waves was registered in athletes who presented with symptoms. In asymptomatic athletes, the tall and flat T-waves were recorded echocardio- graphically. Therefore, young athletes with MVP are often predisposed to electro- cardiographic abnormalities of ventricular repolarization, which requires annual cardiologic evaluation (30).

4) It has to be emphasized that all of the children enrolled had undergone the cochlear implantation under general anesthesia. This means that the cardiac system is able to tolerate a severe stress. It is important to note that some anesthetic drugs, such as sevoflurane, significantly prolong the preoperative QTc (30); which is associated with torsades de pointes. 

In 2011, Niaz et al. evaluated 104 deaf children. One congenital deformity was noted; Patent Ductus Arteriosus. Benign murmurs were detected in 15 deaf-mute children. This study found four patients with a long QT interval (>440 ms). The prevalence of congenital heart disease estimated to be 0.1/1000 (31). 

5) In addition, these children had a family history of congenital heart disease, consanguineous marriage of the parents, twins, low birth weight, pre-term or postdate labor,and NICU admission in their past medical histories, which may be risk factors or an signal for congenital heart disease and require cardiac evaluations.

## Conclusion

Among the 31 children in this study, two had a family history of congenital heart diseases, five had a QTc longer than normal. Two participants had a minimal MR, 15 had MVP, and only one child’s myocardial function was in the minimum range of normal (EF, 56%; FS, 28%). This study shows the possible presence of congenital heart diseases in deaf children. However, further studies are required in order to indicate the necessity of echocardiography and heart examinations in children with SNHL.

Regardless of the severity of the abnormality, the coincidence of these two anomalies can be beneficial for the recognition of the reasons and risk factors. It can be useful for treatment programs specifically cardiac repair surgeries and cochlear implantation, simultaneously.

*Suggestions*

To identify the frequency and prevalence of congenital heart disorders in sensory-neural deaf children, studies in larger samples and a comparison with normal children in the same age range is necessary. These studies could indicate the need for echocardiography and heart examinations in children with SNHL. The necessity of further evaluations in these children in terms of cardiac and other disorders during their lifetime should be clearly described. 
